# Retraction: Research on CO_2_/CH_4_/N_2_ competitive adsorption characteristics of anthracite coal from Shanxi Sihe coal mine

**DOI:** 10.1039/d4ra90145b

**Published:** 2024-12-09

**Authors:** Jia Jinzhang, Xiao Lingyi

**Affiliations:** a College of Safety Science and Engineering, Liaoning Technical University Fuxin Liaoning 123000 China xly010218@163.com; b Key Laboratory of Mine Thermal Power Disaster and Prevention of Ministry of Education, Liaoning Technical University Huludao Liaoning 125100 China

## Abstract

Retraction of ‘Research on CO_2_/CH_4_/N_2_ competitive adsorption characteristics of anthracite coal from Shanxi Sihe coal mine’ by Jia Jinzhang *et al.*, *RSC Adv.*, 2024, **14**, 3498–3512, https://doi.org/10.1039/D3RA08467A.

We, the named authors, hereby wholly retract this *RSC Advances* article due to errors in the simulation results of the paper. After the article was published, we discovered errors in the gas adsorption simulation, and as a result, Fig. 12, 14 and 15 are not accurate enough. The conclusions of this paper are therefore not fully supported.

Signed: Jia Jinzhang and Xiao Lingyi

Date: 28th November 2024

Retraction endorsed by Laura Fisher, Executive Editor, *RSC Advances*

